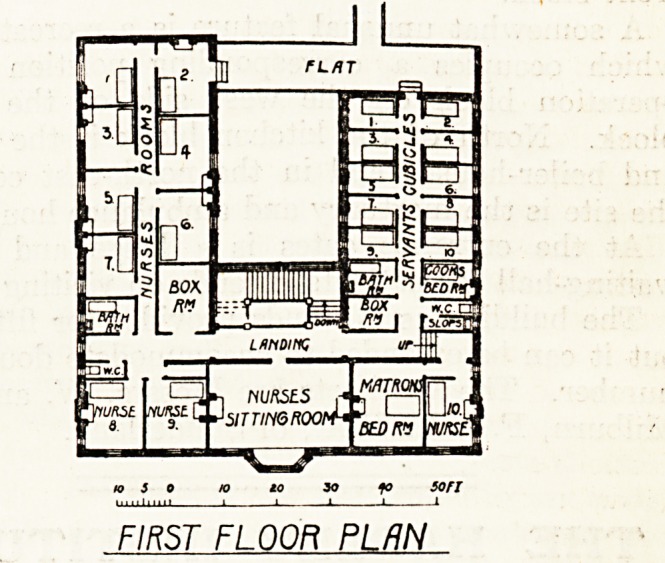# The Children's Hospital, Sunderland, Illustrated with Plans

**Published:** 1913-12-13

**Authors:** 


### HOSPITAL ARCHITECTURE AND CONSTRUCTION.

### The Children's Hospital, Sunderland.

THE building—a complete hospital in itself—is an extension, or rather an addition, to the Royal Infirmary, Sunderland, an institution founded in 1794. The site of the Royal Infirmary being already fully occupied, the governors decided to provide the much-needed extension by building a complete self-contained hospital on a new site.

A site about four acres in extent was acquired on elevated ground, well sheltered from prevailing winds, and with an aspect to the south over a considerable expanse of country. At the present time the site is practically open country, but when the town does expand, its open character cannot be seriously interfered with, as it adjoins a large public park. The building is planned roughly in the form of an E, but with the middle stroke projecting at the back, and with two smaller projections on the same side. The centre building is administration, the two wings being the wards.

The ward blocks have their long axes very nearly north and south, are one storey in height, and are planned very much on the lines of Derbyshire Royal Infirmary. Each ward contains twenty beds, the sanitary offices and bathrooms are placed in projecting wings at the south ends of the wards, and at the entrance end is a two-bed ward, ward kitchen, store cupboards, and lavatory and w.c. for nurses. On the other side of the corridor is a small ward for the isolation of doubtful cases. It would have been a great improvement if the sanitary offices had been all grouped together at the north end of the ward, and the bathroom placed next to the large ward with direct access therefrom. This would have economised the plumbing and hot-water services, and have left the south end of the ward free from obstruction.

The operation suite comprises, besides the theatre, which has a semi-octagonal end, an anæsthetic room, a splint cupboard, and an *x*-ray room. There is apparently no room for sterilising, for washing up, or for the surgeons to prepare for work. The accommodation appears to be strangely meagre and out of date.

FIRST FLOOR PLAN

The administration block is two storeys in height south of the main corridor, one storey only as to the remainder.

In the south part is the main entrance, with committee-room, waiting-room, and matron's sitting-room, and office adjoining. On the east side is an entrance for patients, with waiting-room, receiving-room, and bathroom adjoining, also the charge nurses' sitting-room. On the west are quarters for two resident medical officers. The north wing contains the kitchen offices and stores, and dining-rooms for nurses and servants. The bedrooms for nurses and servants are on the upper floor of the front block.

A somewhat unusual feature is a recreation hall which occupies a corresponding position to the operation block on the west side of the central block. North of the kitchen block is the laundry and boiler-house; and in the north-east corner of the site is the mortuary and ambulance house.

At the entrance gates is a lodge and a large waiting-hall for patients' friends on visiting days.

The building as it stands provides for fifty beds, but it can be extended to accommodate double that number. The architects are Messrs. W. and T. R. Milburn, F.F.R.I.B.A., of Sunderland.

## Figures and Tables

**Figure f1:**
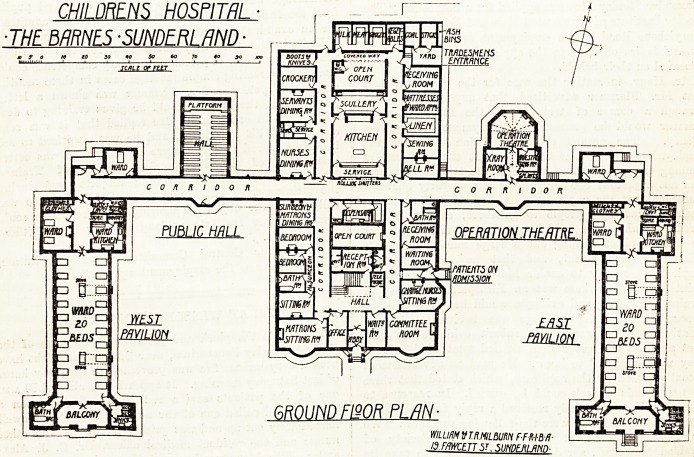


**Figure f2:**